# Supplementary material for: Heterologous immunization with inactivated vaccine followed by mRNA-booster elicits strong immunity against SARS-CoV-2 Omicron variant
Source: Nat Commun. 2022 May 13;13:2670. doi: 10.1038/s41467-022-30340-5 (PMC9106736; doi:10.1038/s41467-022-30340-5)

Supplementary information: Heterologous immunization with inactivated vaccine followed by mRNA-booster elicits strong immunity against SARS-CoV-2 Omicron variant

Fanglei Zuo[1*], Hassan Abolhassani[1*], Likun Du[1*], Antonio Piralla[2*], Federico Bertoglio[3], Leire de Campos-Mata[1], Hui Wan[1], Maren Schubert[3], Irene Cassaniti[2], Yating Wang[1], Josè Camilla Sammartino[2], Rui Sun[1], Stelios Vlachiotis[1], Federica Bergami[2], Makiko Kumagai-Braesch[4], Juni Andréll[5], Zhaoxia Zhang[6], Yintong Xue[7], Esther Veronika Wenzel[3,8], Luigi Calzolai[9], Luca Varani[10], Nima Rezaei[11], Zahra Chavoshzadeh[12], Fausto Baldanti[2,13#], Michael Hust[3#], Lennart Hammarström[1#], Harold Marcotte[1#], Qiang Pan-Hammarström[1#]

[1]*Department of Biosciences and Nutrition, Karolinska Institutet, Huddinge, Sweden.*
[2]*Molecular Virology Unit, Microbiology and Virology Department, Fondazione IRCCS Policlinico San Matteo, Pavia, Italy.*
[3]*Technische Universität Braunschweig, Institute of Biochemistry, Biotechnology and Bioinformatics; Department of Biotechnology, Braunschweig, Germany.*
[4]*Division of Transplantation Surgery, CLINTEC, Karolinska Institutet at Karolinska University Hospital, Stockholm, Sweden.*
[5]*Science for Life Laboratory, Department of Biochemistry and Biophysics, Stockholm University, Stockholm, Sweden.*
[6]*Department of Aging Neurology orthopedics, Karolinska University Hospital Huddinge, Stockholm, Sweden.*
[7]*Department of Immunology, Peking University Health Science Center, Beijing, China.*
[8]*Abcalis GmbH, Science Campus Braunschweig-Süd, Inhoffenstr. 7, 38124 Braunschweig, Germany.*
[9]*European Commission, Joint Research Centre, Ispra, Italy.*
[10]*Institute for Research in Biomedicine, Università della Svizzera italiana (USI), Bellinzona, Switzerland*
[11]*Research Center for Immunodeficiencies, Pediatrics Center of Excellence, Children's Medical Center, Tehran University of Medical Sciences, Tehran, Iran.*
[12]*Pediatric Infections Research Center, Mofid Children's Hospital, Shahid Beheshti University of Medical Sciences, Tehran, Iran.*
[13]*Department of Clinical, Surgical, Diagnostic and Paediatric Sciences, University of Pavia, Pavia, Italy.*

*These authors contributed equally
# These authors jointly supervised this work

Leading contact: qiang.pan-hammarstrom@ki.se

Supplementary Table 1- Demographic data of vaccinated individuals included in this study.

Groups	Number	Male/Female	Median age (IQR), years	Median sampling day after vaccination (IQR)
Before vaccination	12	5/7	37.5 (28.0-42.8)	-
Inactivated vaccine <85 days after 2nd dose	32	18/14	35.5 (30.0-41.2)	49 (23-59)
Inactivated vaccine >85 days after 2nd dose	19	4/15	29.0 (27.0-37.5)	121 (108-157)
Inactivated vaccine <85 days after 3rd dose	6	3/3	56.0 (34.5-67.8)	12 (9-39)
Inactivated vaccine >85 days after 3rd dose	5	3/2	27.0 (26.0-27.0)	92 (91-144)
Inactivated vaccine + 1 dose of mRNA vaccine boosting <85 days	16	6/10	28.0 (26.8-29.0)	27.5 (19.5-37)
mRNA vaccine <85 days after 1st dose	20	8/12	37.5 (27.7-41)	16 (14-19)
mRNA vaccine <85 days after 2nd dose	50	25/25	37.0 (27.5-45.0)	20 (13-36)
mRNA vaccine >85 days after 2nd dose	28	5/23	34.5 (29.8-51.3)	125.5 (101.5-153)
mRNA vaccine <85 days after 3rd dose	40	12/28	44.5 (33.0-53.0)	21 (13-24)
Infected + mRNA vaccine <85 days	8	4/4	49.0 (33.0-51.0)	29 (18-33)

IQR: interquartile range

Supplementary Table 2- Comparison of demographic data and IgG antibody responses against G614-RBD in a subset of individuals selected for NT90 study compared to the main study cohort. Two-sided Fisher's exact test and Chi-square statistic were used for gender comparison and a two-sided Mann-Whitney U test was used for comparison of age and specific-IgG response.

Groups	Male/Female			Median age (IQR), years			Median anti-G614 RBD IgG (BAU/ml)		
	**NT90**	**All**	***P*-value**	**NT90**	**All**	***P*-value**	**NT90**	**All**	***P*-value**
Before vaccination	4/2	5/7	0.31	38.0 (37.3-39.5)	37.5 (28.0-42.8)	0.77	10.3 (7.9-13.7)	8.9 (6.0-12.5)	0.66
Inactivated vaccine < 85 days after 2nd dose	3/5	18/14	0.34	29.5 (28.8-34.0)	35.5 (30.0-41.2)	0.06	116.1 (39.9-357.0)	338.7 (182.4-690.7)	0.05
Inactivated vaccine > 85 days after 2nd dose	3/6	4/15	0.64	27.0 (24.0-29.0)	29.0 (27.0-37.5)	0.10	25.8 (14.8-44.3)	123.5 (26.6-356.6)	0.06
Inactivated vaccine < 85 days after 3rd dose	3/2	3/3	0.74	67.0 (45.0-68.0)	56.0 (34.5-67.8)	0.84	295.0 (170.4-385.2)	308.2 (201.6-369.3)	0.97
Inactivated vaccine > 85 days after 3rd dose	2/0	3/2	1.0	26.0 (25.5-26.5)	27.0 (26.0-27.0)	0.76	27.2 (24.7-29.7)	32.7 (26.6-54.3)	0.61
Inactivated vaccine + 1 dose of mRNA vaccine boosting < 85 days	5/8	6/10	0.95	28.0 (26.0-29.0)	28.0 (26.8-29.0)	0.95	2230.4 (1336,0-4306.0)	2121.0 (1313.2-3471.5)	0.82
mRNA vaccine < 85 days after 2nd dose	5/4	25/25	0.78	38.0 (34.0-41.0)	37.0 (27.5-45.0)	0.46	1001.2 (914.3-1281.8)	1229.4 (745.8-2408.4)	0.54
mRNA vaccine > 85 days after 2nd dose	4/4	5/23	0.07	39.0 (36.3-44.0)	34.5 (29.8-51.3)	0.32	282.0 (218.4-339.8)	245.8 (137.1-422.9)	0.84
mRNA vaccine < 85 days after 3rd dose	6/18	12/28	0.66	48.5 (38.0-53.8)	44.5 (33.0-53.0)	0.36	2128.4 (1280.0-3063.5)	2239.0 (1380.2-4063.8)	0.68
Infected + mRNA vaccine < 85 days	5/3	5/3	1.0	43.0 (32.3-49.5)	49.0 (33.0-51.0)	0.81	2071.8 (1329.0-2504.6)	2104.7 (1416.5-2608.8)	0.74

IQR: interquartile range

Supplementary Fig.1. Study design of the four main groups of 175 vaccinated individuals including homologous mRNA, homologous inactivated, heterologous vaccinated and infected vaccinated individuals. Blue and red circles show the mRNA and inactivated vaccine doses, respectively. "S" indicated the samples available for this study. Dark line depicts individuals with multiple samples before and/or after vaccinations.

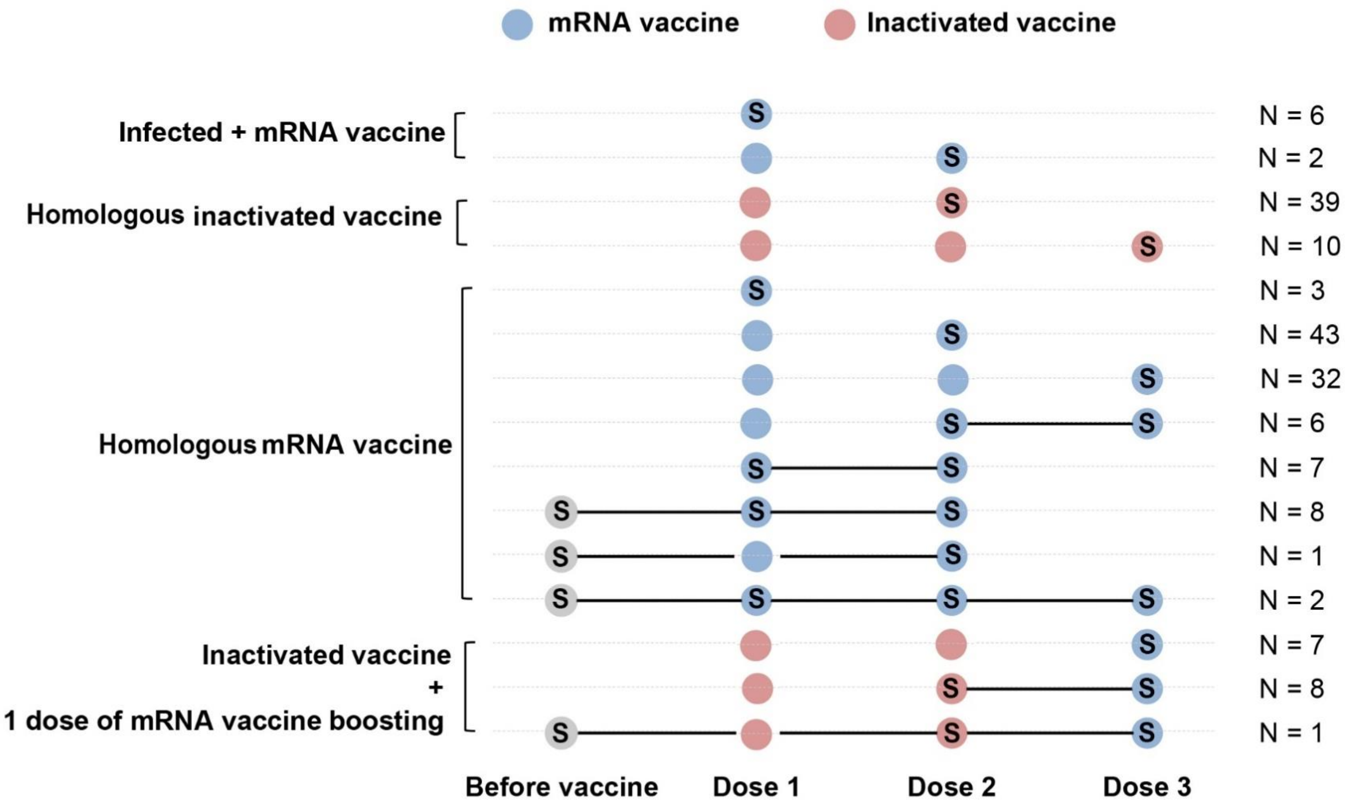

Supplementary Fig.2. Comparison of specific IgG antibody response against receptor-binding domain (RBD) of SARS-CoV-2 G614 in individuals within groups of mRNA vaccines (a), inactivated vaccines (b) presenting as binding antibody units (BAU)/ml. Whiskers indicate the interquartile range. Two-sided Mann-Whitney U test was used and P < 0.05 was considered statistically significant and marked with red color.

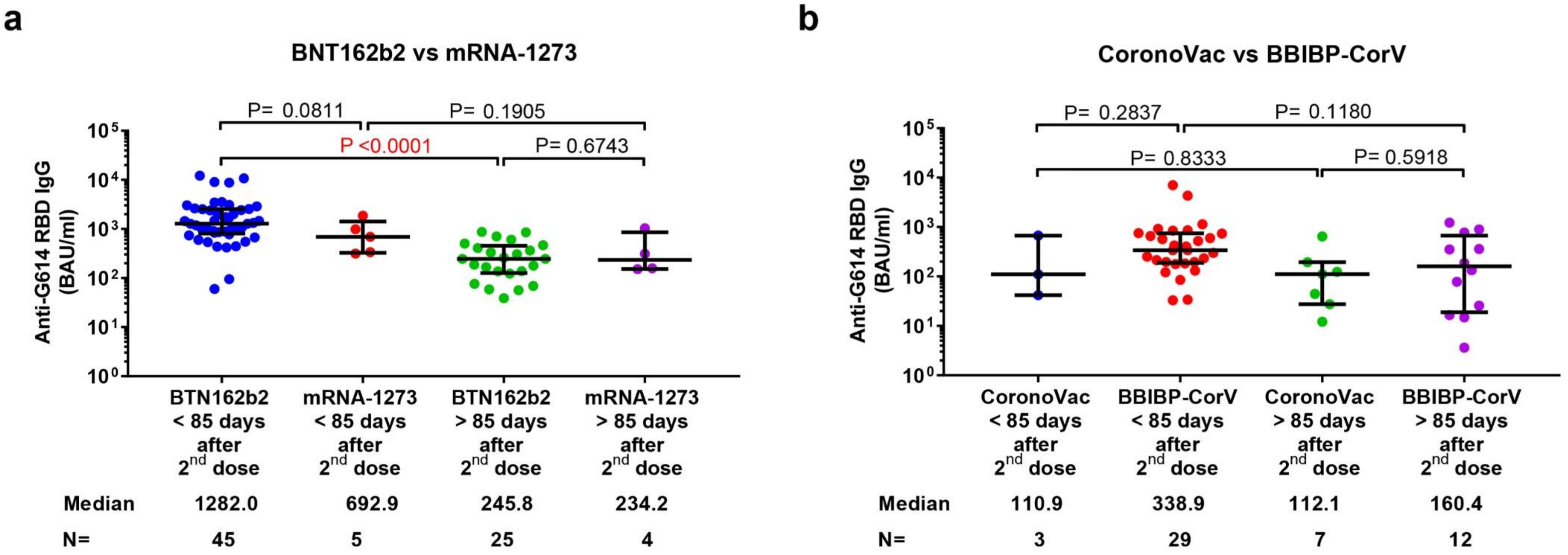

Supplementary Fig.3. Decline of antibody levels against receptor-binding domain (RBD) presenting as binding antibody units (BAU)/ml with time in different vaccine groups. Inactivated/mRNA prime-boost vaccination compared to (a) two doses of the homologous inactivated or mRNA vaccines, (b) three doses of the homologous inactivated or mRNA vaccines.

a.

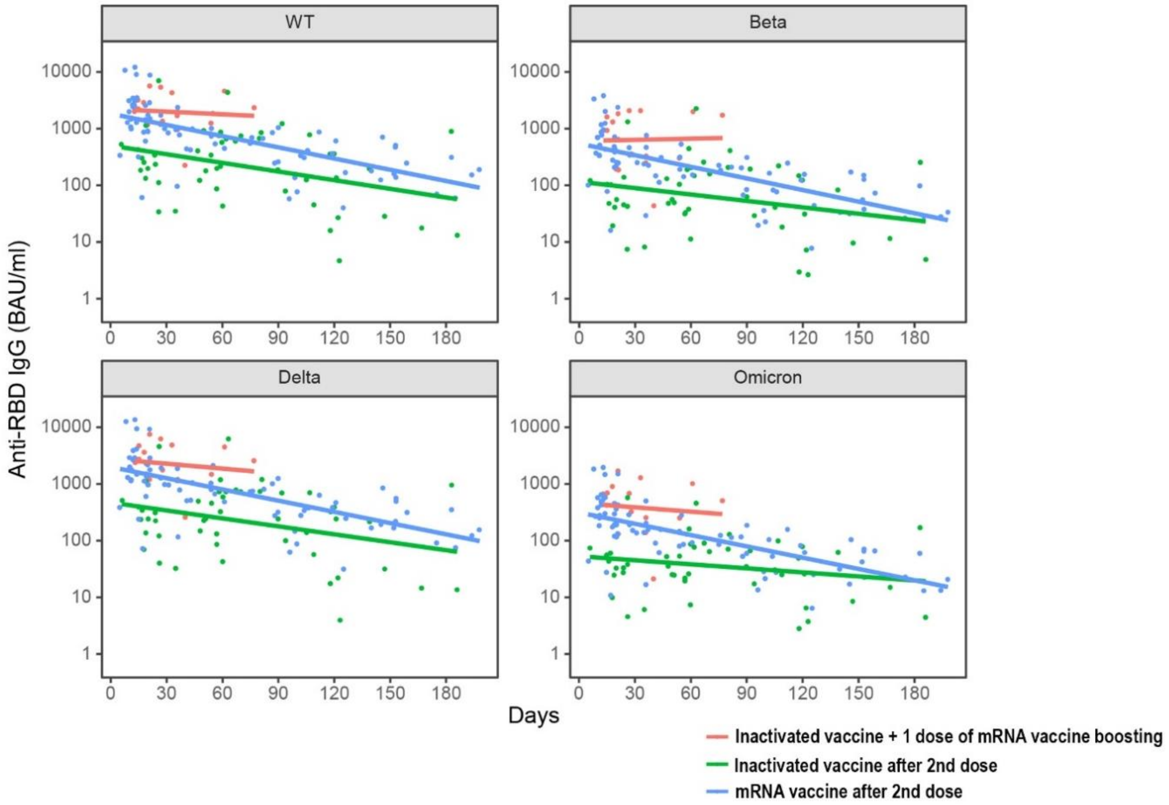

b.

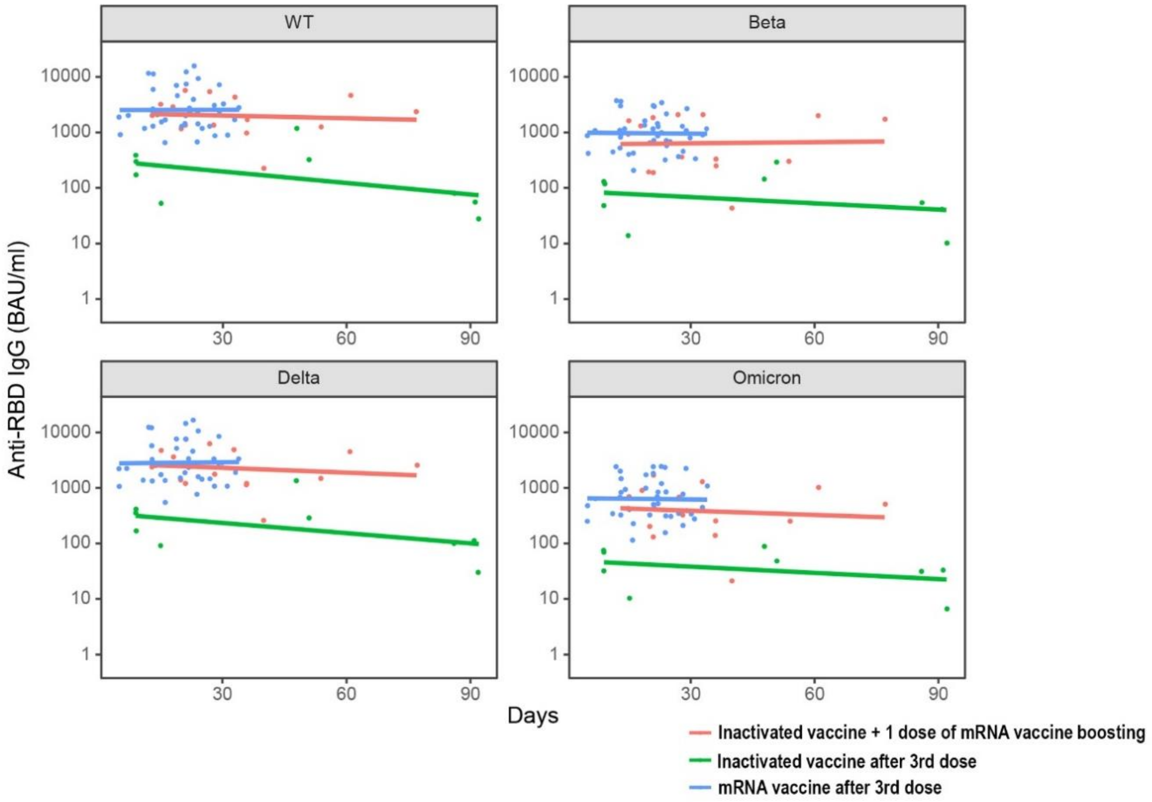

Supplementary Fig.4. Longitudinal analysis of antibody responses against receptor-binding domain (RBD) of G614 SARS-CoV-2 and variants of concern in different groups of vaccinated individuals presenting as binding antibody units (BAU)/ml. Symbols represent individual subjects. The cutoff-value (dashed red line) is indicated.

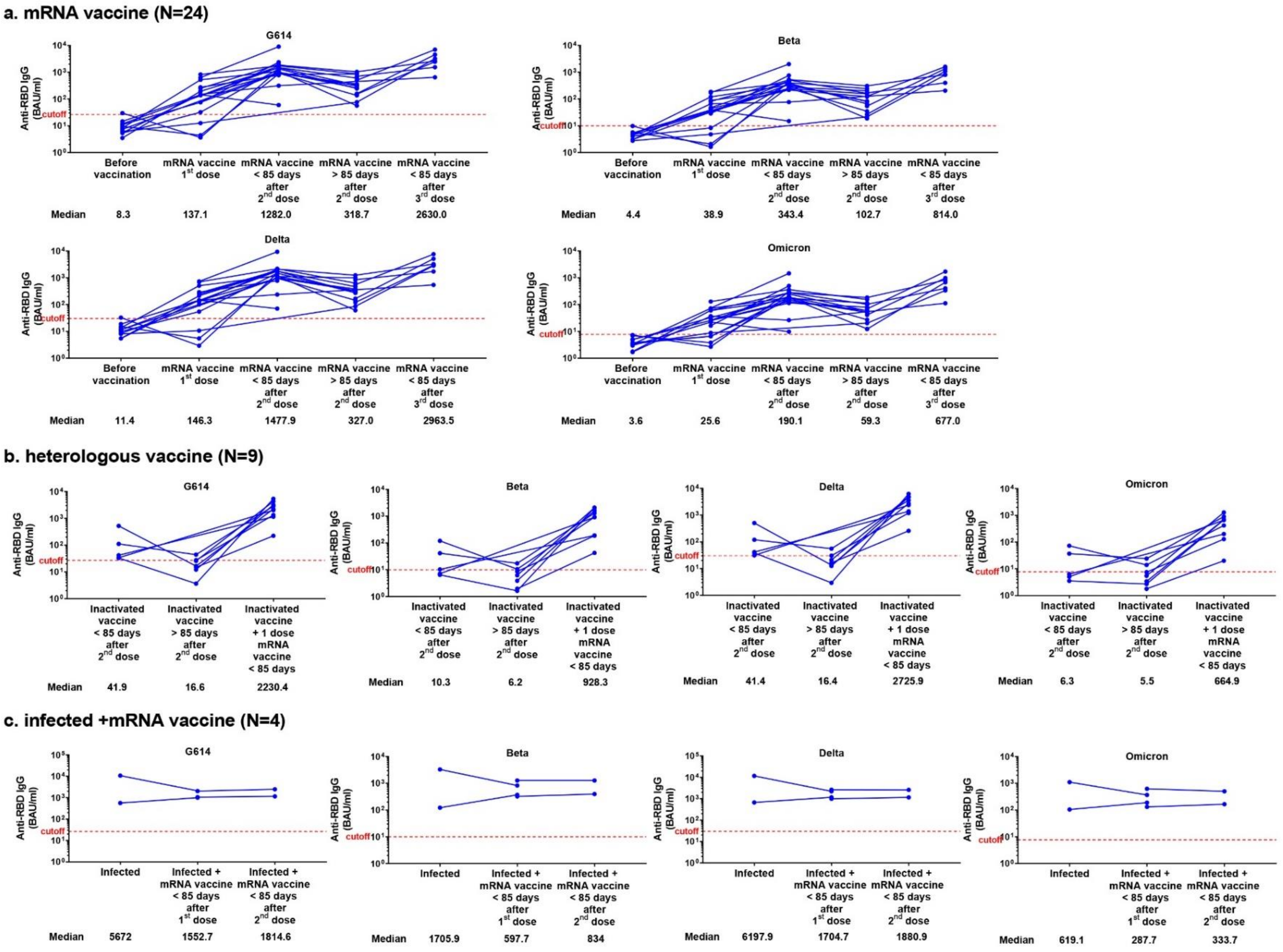

Supplementary Fig.5. Anti- receptor-binding domain (RBD) IgG responses against Beta, Delta and Omicron variants of concern compared to G614 presenting as binding antibody units (BAU)/ml. Symbols represent individual subjects and horizontal black lines indicate the median. The number of fold differences of median in comparison to G614 is shown. Naturally infected individuals in the last group were color-coded based on receiving one (7 samples) or two doses (3 samples) of mRNA vaccines. Whiskers indicate the interquartile range. Two-sided Mann-Whitney U test was used and P < 0.05 was considered statistically significant and marked with red color.

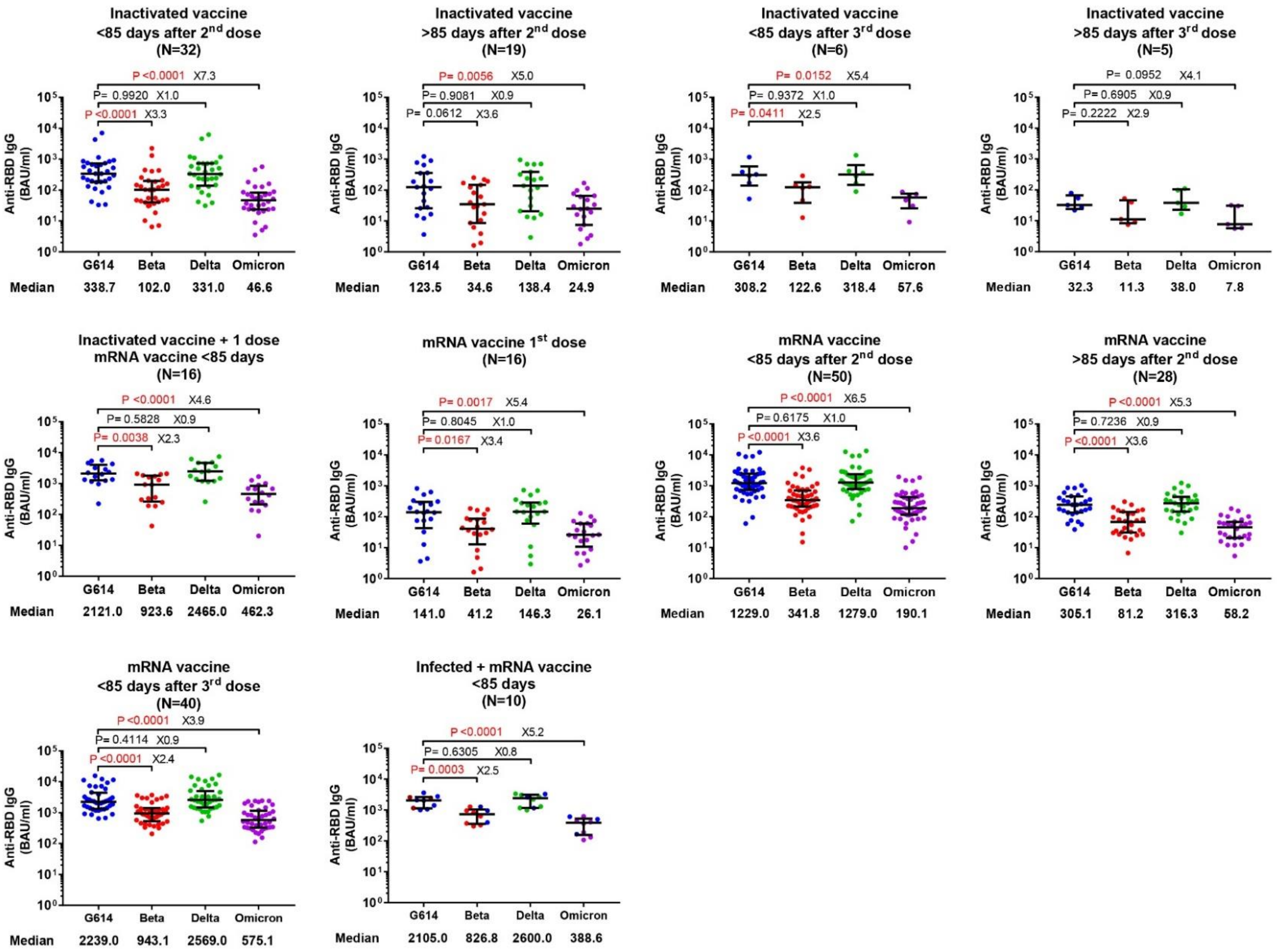

Supplementary Fig.6. IgG antibody response against the spike (S) protein of G614 and Omicron in different groups of vaccinated individuals presenting as binding antibody units (BAU)/ml. Symbols represent individual subjects and horizontal black lines indicate the median. The cutoff-value (dashed red line) and number of fold differences of median between groups are indicated. For each group, the number of samples (N=) and median antibody titers are shown below the X-axis. In the last group, convalescent donors (prior history of infection) were color-coded based on receiving one (purple) or two doses (blue) of mRNA vaccines. Whiskers indicate the interquartile range. Two-sided Mann-Whitney U test was used and P < 0.05 was considered statistically significant and marked with red color.

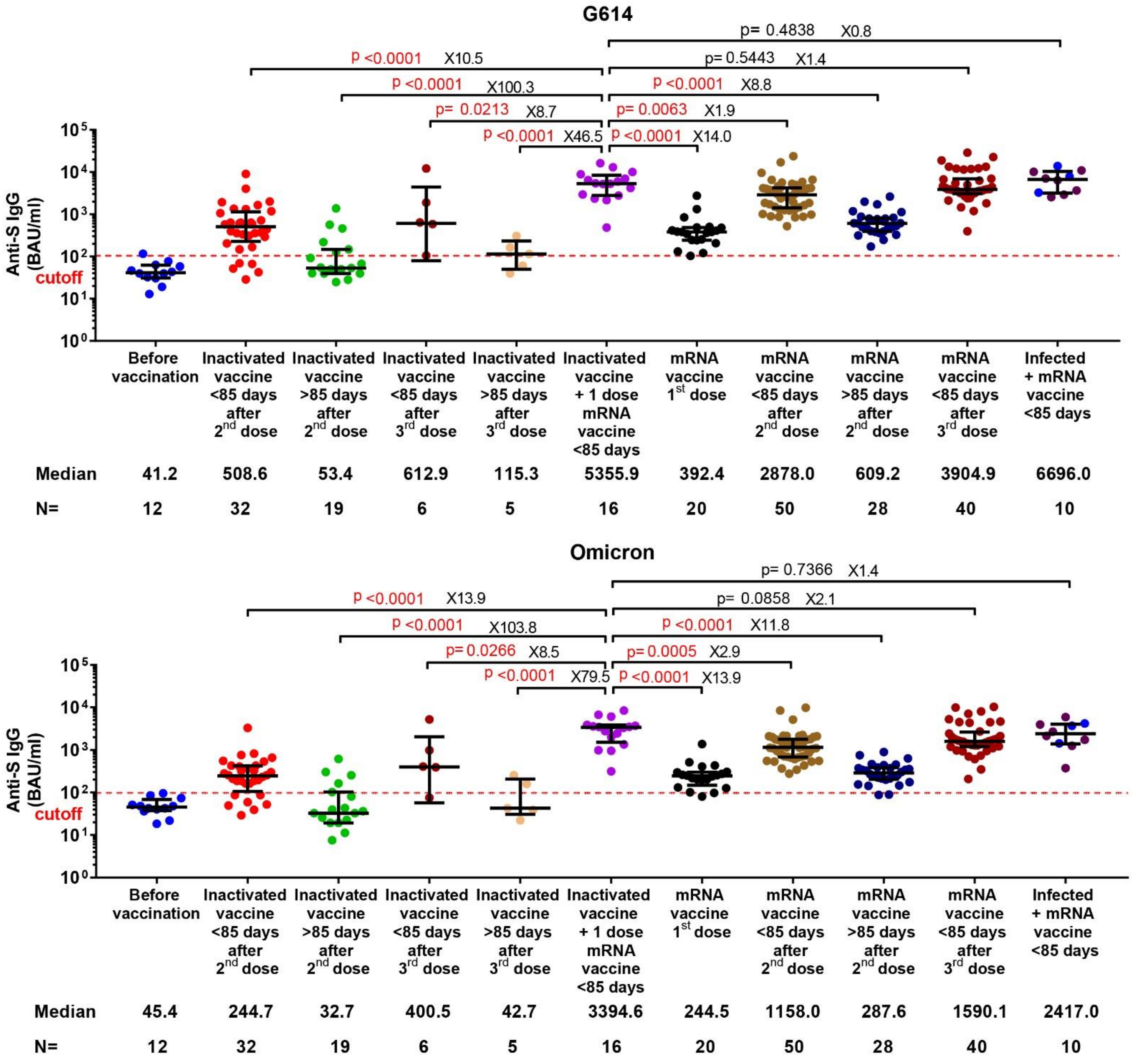

Supplementary Fig.7. Comparison of antibody responses against spike (S) protein of G614 and Omicron in different groups of vaccinated individuals presenting as binding antibody units (BAU)/ml. Symbols represent individual subjects and horizontal black lines indicate the median. The number of fold differences of median between groups is indicated. For each group, median antibody titers are shown below the X-axis. In the last group, convalescent donors (prior history of infection) were color-coded based on receiving two doses of mRNA vaccines in purple. Whiskers indicate the interquartile range. Two-sided Mann-Whitney U test was used and P < 0.05 was considered statistically significant and marked with red color.

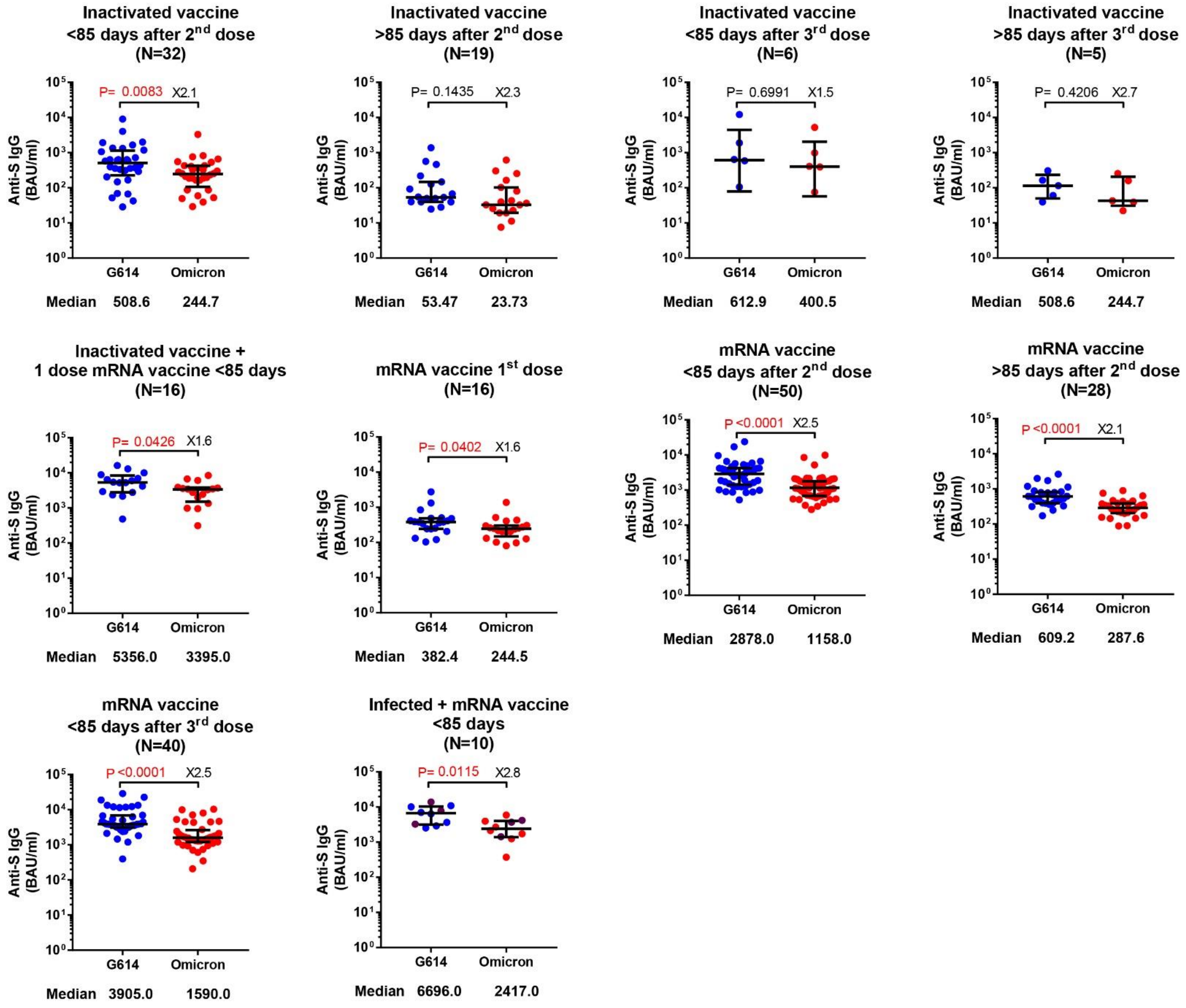

Supplementary Fig.8. Spike protein (S), nucleoprotein (N), membrane protein (M), and open reading frame (ORF)-3a and ORF-7a proteins (O) peptide (SNMO) pool-specific T cell responses in different groups of vaccinated individuals. Symbols represent individual subjects and horizontal black lines indicate the median. The cutoff-value (dashed red line) and number of fold differences of median between groups are indicated. For each group, the number of samples (N=) and median number of specific T cells are shown below the X-axis. Naturally infected individuals were color-coded based on receiving one (purple) or two doses (blue) of mRNA vaccines. Whiskers indicate the interquartile range. Two-sided Mann-Whitney U test was used and P < 0.05 was considered statistically significant and marked with red color. IL-2: interleukin 2, IFN-γ: Interferon gamma.

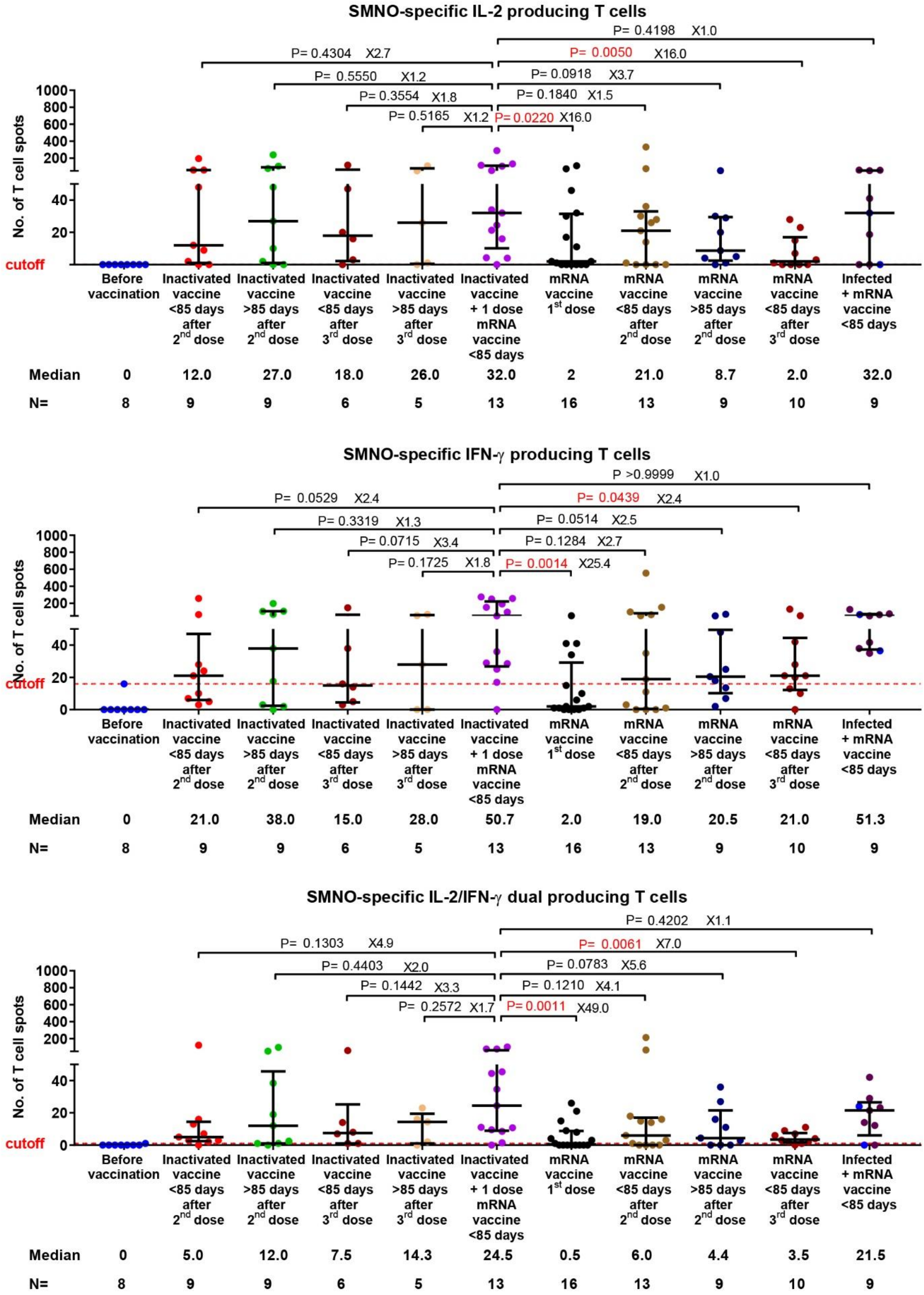

Supplement: Supplementary file 1 — Supplementary information [file 41467_2022_30340_MOESM1_ESM.pdf]
