# Supplementary material for: Heterologous immunization with inactivated vaccine followed by mRNA-booster elicits strong immunity against SARS-CoV-2 Omicron variant
Source: Nat Commun. 2022 May 13;13:2670. doi: 10.1038/s41467-022-30340-5 (PMC9106736; doi:10.1038/s41467-022-30340-5)
Supplement: Supplementary file 3 — Reporting Summary [file 41467_2022_30340_MOESM3_ESM.pdf]

nature portfolio

Corresponding author(s): Qiang Pan Hammarström
Last updated by author(s): Apr 8, 2022

Reporting Summary

Nature Portfolio wishes to improve the reproducibility of the work that we publish. This form provides structure for consistency and transparency in reporting. For further information on Nature Portfolio policies, see our Editorial Policies and the Editorial Policy Checklist.

Statistics

For all statistical analyses, confirm that the following items are present in the figure legend, table legend, main text, or Methods section.

n/a	Confirmed	
☐	☒	The exact sample size (n) for each experimental group/condition, given as a discrete number and unit of measurement
☐	☒	A statement on whether measurements were taken from distinct samples or whether the same sample was measured repeatedly
☐	☒	The statistical test(s) used AND whether they are one- or two-sided<br>*Only common tests should be described solely by name; describe more complex techniques in the Methods section.*
☐	☒	A description of all covariates tested
☒	☐	A description of any assumptions or corrections, such as tests of normality and adjustment for multiple comparisons
☐	☒	A full description of the statistical parameters including central tendency (e.g. means) or other basic estimates (e.g. regression coefficient) AND variation (e.g. standard deviation) or associated estimates of uncertainty (e.g. confidence intervals)
☐	☒	For null hypothesis testing, the test statistic (e.g. F, t, r) with confidence intervals, effect sizes, degrees of freedom and P value noted<br>*Give P values as exact values whenever suitable.*
☒	☐	For Bayesian analysis, information on the choice of priors and Markov chain Monte Carlo settings
☒	☐	For hierarchical and complex designs, identification of the appropriate level for tests and full reporting of outcomes
☒	☐	Estimates of effect sizes (e.g. Cohen's d, Pearson's r), indicating how they were calculated

Our web collection on statistics for biologists contains articles on many of the points above.

Software and code

Policy information about availability of computer code

Data collection	Microsoft Excel 2017 was used for data collection for this study.
Data analysis	GraphPad version 7.05 or R version 3.6.1. were used for data analysis and the statistical analysis tests are cited in the "Methods" section.

For manuscripts utilizing custom algorithms or software that are central to the research but not yet described in published literature, software must be made available to editors and reviewers. We strongly encourage code deposition in a community repository (e.g. GitHub). See the Nature Portfolio guidelines for submitting code & software for further information.

Data

Policy information about availability of data

All manuscripts must include a data availability statement. This statement should provide the following information, where applicable:
- Accession codes, unique identifiers, or web links for publicly available datasets
- A description of any restrictions on data availability
- For clinical datasets or third party data, please ensure that the statement adheres to our policy

All data used to support the findings are included in the article or in the supplementary data and coresponding data for each figures have been provided in Source Data file separately and all raw data supporting the findings of this study are available online in Zenodo at ://zenodo.org/record/6305550#.YhzpWmRKhaQ..

Field-specific reporting

Please select the one below that is the best fit for your research. If you are not sure, read the appropriate sections before making your selection.

[x] Life sciences   [ ] Behavioural & social sciences   [ ] Ecological, evolutionary & environmental sciences

For a reference copy of the document with all sections, see nature.com/documents/nr-reporting-summary-flat.pdf

Life sciences study design

All studies must disclose on these points even when the disclosure is negative.

Sample size	All 238 samples from 175 healthy volunteers in different vaccination groups were recruited during 2021-2020. As this was an observational study no sample size analysis was performed. However, to the best of our knowledge this is the largest dataset evaluating both the humoral and cellular immune response in heterologous vaccinated individuals with inactivated and mRNA vaccines.
Data exclusions	None. As mentioned above all 238 samples tested during the study period.
Replication	Replication have been conducted at the time of immunologic method setup, conferring a robust results for all studied samples. The experiments were replicated 10 times independently and we confirm that all attempts at replication were successful.
Randomization	Randomization was not applicable for this study since we include "all" volunteers prospectively in each group of vaccination.
Blinding	We anonymized specimens and labeled specific code for each studied sample therefore handling colleagues who performed the experiment worked with coded samples and were unable to recognize the vaccination groups or infection status.

Reporting for specific materials, systems and methods

We require information from authors about some types of materials, experimental systems and methods used in many studies. Here, indicate whether each material, system or method listed is relevant to your study. If you are not sure if a list item applies to your research, read the appropriate section before selecting a response.

Materials & experimental systems

n/a	Involved in the study	
[ ]	[x]	Antibodies
[x]	[ ]	Eukaryotic cell lines
[x]	[ ]	Palaeontology and archaeology
[x]	[ ]	Animals and other organisms
[ ]	[x]	Human research participants
[x]	[ ]	Clinical data
[x]	[ ]	Dual use research of concern

Methods

n/a	Involved in the study	
[x]	[ ]	ChIP-seq
[x]	[ ]	Flow cytometry
[x]	[ ]	MRI-based neuroimaging

Antibodies

Antibodies used	Detection of antibodies specific to SARS-CoV-2: horseradish peroxidase (HRP)-conjugated goat anti-human IgG (Invitrogen #A18805)<br>the Human IgG SARS-CoV-2 RBD ELISpotPLUS kit (mabtech#3850-4HPW-R1-1) contains anti-WASP-HRP, anti-MT78/145<br>the Human IFNg-IL2 SARS-CoV-2 FluroSpot plus kit (mabtech# FSP-0102-P1-1) contains anti-IFN-γ, anti-IL-2, anti-CD3, anti-CD28
Validation	All antibodies used are commercially available and were validated by the manufacturer with the details available on respective websites, the link for which are provided as follows:<br>https://www.thermofisher.com/antibody/product/Goat-anti-Human-IgG-H-L-Secondary-Antibody-Polyclonal/A18805<br>https://www.mabtech.com/products/elispot-path-human-igg-sars-cov-2-rbd-hrp-3850-4hpw-r1<br>https://www.mabtech.com/products/fluorospot-path-human-ifn-gamma-il-2-sars-cov-2-s1scansnmo_fsp-0102-p1-1

Human research participants

Policy information about studies involving human research participants

Population characteristics	The study includes 238 samples from 175 healthy volunteers (58.8% females, median age of 36 years) in Sweden (n=101), Germany (n=18), Iran (n=34), and Italy (n=22) recruited during 2021-2022. Individuals were followed at 1 (n=142), 2 (n=22), 3 (n=9) or 4 time points (n=2) during their respective vaccination schedule (Supplementary Fig1). The samples were further characterized based on the vaccination record: homologous inactivated vaccination (BBIBP-CorV, n= 42, 45 samples; CoronoVac, n=7, 8 samples), homologous mRNA vaccination (BNT162b2, n=94, 123 samples; mRNA-127, n=8, 15 samples), heterologous vaccination with two doses of inactivated vaccine followed by an mRNA vaccine boost at 4-16 months (n=16, 9 samples before and 16 samples after booster), and homologous mRNA vaccination preceded by a prior history of mild SARS-CoV-2 infection based on self-reported or laboratory evidence (n=8, 10 samples). Serum samples from pre-vaccinated, non-infected healthy donors from our cohort (n=12) were also collected as negative controls.
Recruitment	Recruitment criteria for this study was. 18 years of age and older. Able to read, speak, and understand English. Able to provide informed consent. In current good general health.<br><br>Potential biases for this study include a rather low number of total participants and limited access to extensive prospective sample collection. The volunteers included in the study also tend to be younger than the average global population. Furthermore, the data from various vaccination groups were mainly compared using cross-sectional analysis and longitudinal analysis was performed only in a subset of samples.
Ethics oversight	The study was approved by the ethics committees in institutional review board (IRB) of Stockholm, Technische Universität Braunschweig, the Tehran University of Medical Sciences, and the Policlinico San Matteo.

Note that full information on the approval of the study protocol must also be provided in the manuscript.
